# Effect of *p22phox* depletion on sympathetic regulation of blood pressure in SHRSP: evaluation in a new congenic strain

**DOI:** 10.1038/srep36739

**Published:** 2016-11-08

**Authors:** Hasan M. Zahid, Mohammed Zubaerul Ferdaus, Hiroki Ohara, Minoru Isomura, Toru Nabika

**Affiliations:** 1 Department of Functional Pathology, Shimane University School of Medicine, Izumo, Japan

## Abstract

Oxidative stress in the rostral ventrolateral medulla (RVLM), a sympathetic center in the brainstem, was implicated in the regulation of sympathetic activity in various hypertensive models including stroke-prone spontaneously hypertensive rats (SHRSP). In this study, we evaluated the role of the NADPH oxidases (NOX) in the blood pressure (BP) regulation in RVLM in SHRSP. The P22PHOX-depleted congenic SHRSP (called SP.MES) was constructed by introducing the mutated *p22phox* gene of Matsumoto Eosinophilic Shinshu rat. BP response to glutamate (Glu) microinjection into RVLM was compared among SHRSP, SP.MES, SHR and Wistar Kyoto (WKY); the response to Glu microinjection was significantly greater in SHRSP than in SP.MES, SHR and WKY. In addition, tempol, losartan and apocynin microinjection reduced the response to Glu significantly only in SHRSP. The level of oxidative stress, measured in the brainstem using lucigenin and dihydroethidium, was reduced in SP.MES than in SHRSP. BP response to cold stress measured by telemetry system was also blunted in SP.MES when compared with SHRSP. The results suggested that oxidative stress due to the NOX activation in RVLM potentiated BP response to Glu in SHRSP, which might contribute to the exaggerated response to stress in this strain.

Reactive oxygen species (ROS) have been implicated in various cardiovascular diseases including hypertension. Among diverse effects of ROS in the pathogenesis of hypertension, the regulatory role in the sympathetic nervous system attracted our attention since the sympathetic nervous system plays an important role in the pathogenesis of hypertension^1^. Several studies indicated that an increased ROS level in the rostral ventrolateral medulla (RVLM), one of the most important regulatory centers of the sympathetic nerve activity, was observed in various hypertensive models including the spontaneously hypertensive rat (SHR) and the stroke-prone SHR (SHRSP)^2^,^3^,^4^,^5^.

ROS are regulated by a combination of several different pathways that either produce or degrade ROS^3^, and thus it is difficult to understand roles of particular pathway(s) on the regulation of blood pressure (BP). Among pathways producing ROS, NADPH oxidases (NOX) are known to play an important role in various pathophysiological processes including hypertension^3^. Indeed, over-expression of several NOX subunits such as NOX2, P22PHOX, NOX1 and NOX4 were observed when arterial cells were stimulated with angiotensin II (AngII)^6^. In this study, we therefore hypothesized that NOX were responsible for the high level of ROS in RVLM in SHRSP, which might causally relate to the exaggerated sympathetic responsiveness in this model rat. Therefore our aim was to clarify roles of NOX in the sympathetic regulation of BP in SHRSP.

To examine this hypothesis, we introduced a P22PHOX-depleted congenic SHRSP, which was recently developed in our laboratory^7^. This congenic strain (called as SP.MES) was established to harbor the null mutation in the *P22phox* gene of the Matsumoto Eosinophilic Shinshu rat (MES)^8^. P22PHOX is a membrane-bound subunit that is essential for the NOX activity, and therefore it was expected that the most of the NOX activity was depleted in SP.MES.

In this communication, we showed that the response to glutamate (Glu) injection into RVLM differed significantly between SP.MES and SHRSP, suggesting the key role of NOX in sympathetic regulation of BP in SHRSP.

## Results

Table 1 summarizes baseline BP and body weight (BW) of the strains used in the study. SP.MES showed significantly lower BP than that in SHRSP and PM0 [a congenic reference strain that was employed as the control for SP.MES (see Materials and Methods)], while it was significantly higher than that in WKY. BW of SP.MES was less than that in the other four strains.

### Response to Glu microinjection

**Figure 1:**
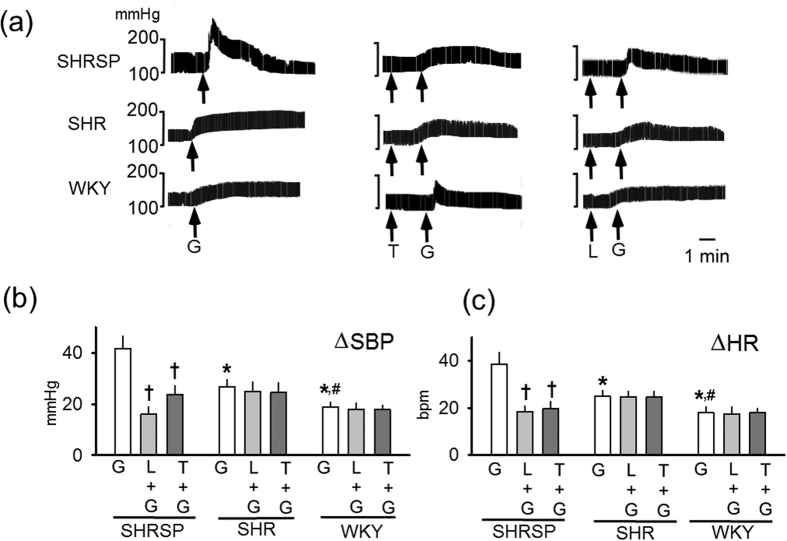
Response to Glu microinjection in SHRSP, SHR and WKY. (**a**) Representative recordings of response to Glu microinjection. (**b**,**c**) Increase in SBP and in HR on Glu microinjection, respectively. Numbers of rats used in the experiments were 5 SHRSP, 5 SHR and 4 WKY in each experiment. *Significantly different when compared with the response in SHRSP. ^†^Significantly different from the response to glutamate (G) alone. ^#^Significantly different from the response in SHR. The Bonferroni’s post-hoc test was applied for the analyses. G: glutamate, L: losartan, T: tempol.

Baseline BP under anesthesia differed significantly among SHRSP, SHR and WKY (162 ± 5, 142 ± 4 and 105 ± 4 mmHg, respectively, p < 0.05 by the Bonferroni post-hoc test). Microinjection of Glu into RVLM elicited a greater increase of BP and heart rate (HR) in SHRSP when compared with SHR and WKY (Fig. 1). Losartan, a blocker of the Ang II receptor type I (AT1R), as well as tempol, a ROS scavenger, reduced the exaggerated response in SHRSP, while no further reduction was observed when these reagents were injected into SHR and WKY (Fig. 1). These results implied that the exaggerated response to Glu observed in SHRSP was due to activation of ROS production, which might be regulated by AT1R.

**Figure 2:**
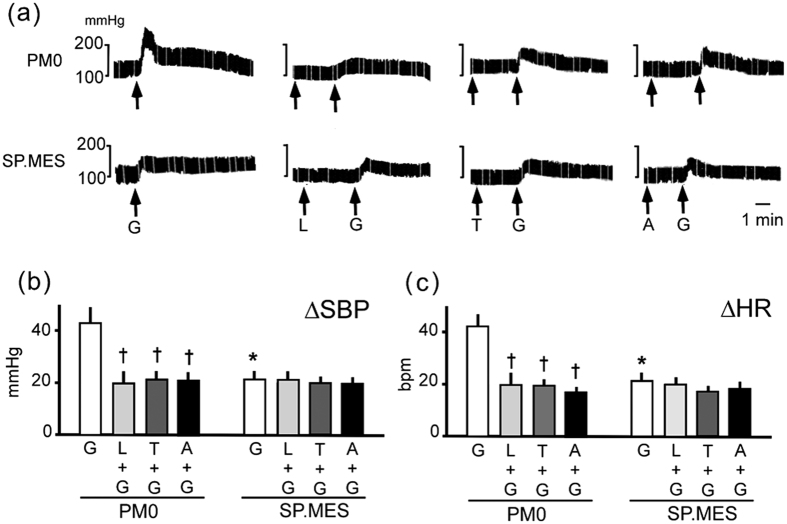
Response to Glu microinjection in PM0 and SP.MES. (**a**) Representative recordings of response to Glu microinjection. (**b**,**c**) Increase in SBP and in HR on Glu microinjection, respectively. Numbers of rats used in the experiments were; 14 PM0 and 11 SP.MES for Glu alone (G), 7 PM0 and 7 SP.MES for losartan + Glu (L+G), 8 PM0 and 5 SP.MES for tempol + Glu (T+G), and 7 PM0 and 5 SP.MES for apocynin + Glu (A+G). *Significantly different when compared with PM0. ^†^Significantly different when compared with the response to Glu alone. The Bonferroni’s post-hoc test was applied for the analyses. G: Glu, T: tempol, L: losartan, A: apocynin.

The role of NOX in the response to Glu was inferred in the experiments using the P22PHOX-depleted SP.MES (Fig. 2). Baseline BP under anesthesia was higher in PM0 compare to SP.MES (158 ± 5 and 133 ± 4 mmHg for PM0 and SP.MES, respectively, p<0.05 by the Student’s t-test). Glu microinjection elicited a greater increase of BP and HR in PM0 when compared with SP.MES (Fig. 2). Losartan, tempol and apocynin (an inhibitor for NOXs; although it has not yet been reported how apocynin is metabolized to exert the inhibitory action in RVLM, apocynin is widely used to inhibit NOX in the RVLM^9^) significantly reduced the response in PM0 to the level of that in SP.MES. In contrast, no further changes were observed in SP.MES when either of the three reagents were injected (Fig. 2).

Although apocynin was reported to have actions in addition to NOX inhibition^10^,^11^, the effect of this compound on the BP response was probably through inhibition of NOX because the P22PHOX-depleted SP.MES mimicked the effect of apocynin.

### ROS level in the brainstem

**Figure 3:**
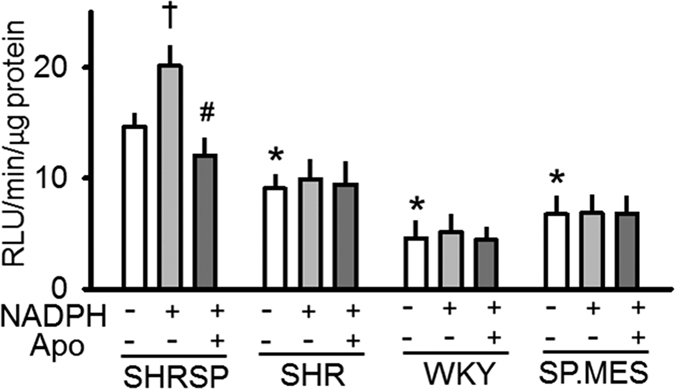
ROS production in the brainstem of SHRSP, SHR, WKY and SP.MES. ROS production in the brainstem was quantified by the lucigenin-enhanced chemiluminescence method as described in Methods. Numbers of rats used were 7 PM0, 5 SHR, 4 WKY and 5 SP.MES. *Significantly different when compared with SHRSP. ^†^Significantly different from the baseline level [i.e., NADPH (−) and Apo (−)]. ^#^Significantly different when compared with the level under NADPH (+).The Bonferroni’s post-hoc test was applied for the analyses. Apo; apocynin, RLU; relative light unit.

ROS production in the brainstem quantified by the lucigenin method was greater in SHRSP than in SHR and WKY. Addition of NADPH, the substrate of NOX, elicited a significant increase in the ROS production that was abolished with apocynin in SHRSP. As the response to NADPH and apocynin in SHR and WKY were less clear than those in SHRSP, the activity of the NOX system in the brainstem seemed greater in SHRSP than in the other two strains (Fig. 3). This result was further supported by the observation in SP.MES; the ROS level in this congenic strain was significantly less than that in SHRSP and was comparable with that in SHR and WKY (Fig. 3). As SHR as well as SP.MES was hypertensive when compared with WKY even though the NOX activity seemed low in these 3 strains (Table 1 and Fig. 3), hypertension in SHR (and SP.MES) seemed less dependent on ROS generation by NOX.

In the ROS measurement using lucigenin, relatively high level of ROS production was observed at a baseline condition as well as no or small increase of ROS after addition of NADPH. This might be due to our experimental condition in which crude cell lysate was used. In this condition, endogenous NADPH might influence the measurement. In spite of that, exogenous NADPH was added to obtain the maximal NOX activity and therefore the difference of the NOX activity after addition of NADPH reflected the difference in the maximal NOX activity among the strains.

To confirm the difference, DHE staining of the RVLM region was performed which gave a result supportive of that in the lucigenin experiment (see Fig. S1).

### Response to cold stress

**Figure 4:**
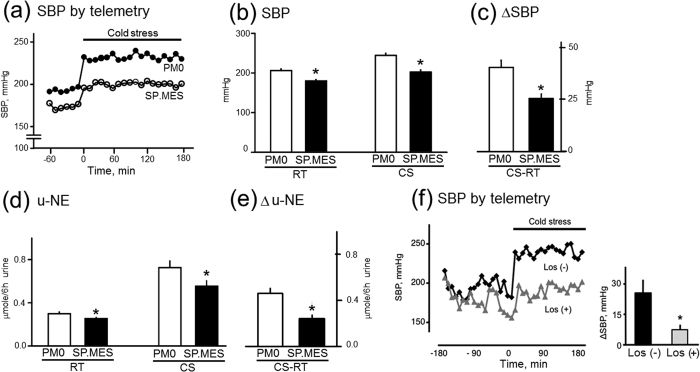
Effects of cold stress in PM0 and SP.MES. Effects of cold stress on SBP: Numbers of rats used were 5 PM0 and 3 SP.MES. (**a**) The time course of BP changes is shown (**b**) SBP at room temperature (RT) and under cold stress (CS) are summarized (**c**) increase in SBP under cold stress (ΔSBP). *Significantly lower than SBP in PM0. Effects of cold stress on u-NE: Numbers of rats used are 7 PM0 and 5 SP.MES. (**d**) u-NE at room temperature (RT) and under cold stress (CS) are summarized (**e**) increase in u-NE under cold stress (Δu-NE). *Significantly different from the level in PM0. RT; room temperature, CS; cold stress, u-NE; urinary norepinephrine, SBP; systolic blood pressure. (**f**) Effect of losartan on response to cold stress in SHRSP. Response to cold stress was examined with and without losartan infused into the lateral ventricle. Numbers of rats used were 3 in each experiment. *Significantly different from Los (−). Los: losartan. The Student’s t-test was applied in the analyses.

Previous studies suggested that Ang II and ROS played an important role in stress-induced sympathetic activation^12^,^13^. In this study, the response to cold stress was therefore examined in SP.MES to evaluate a role of NOX in the stress response. Baseline BP measured by the telemetry was lower in SP.MES than in SHRSP (Fig. 4a,b,c). Under the cold stress, increase in SBP was significantly smaller in SP.MES than in SHRSP (Fig. 4a,c). Further, increase of urinary norepinephrine (u-NE) under the stress was significantly less in SP.MES (Fig. 4d,e). These observations indicated that BP increase due to sympathetic activation under cold stress was attenuated in SP.MES. In addition, infusion of losartan into the lateral ventricle decreased the response to cold stress in SHRSP, supporting the important role of Ang II in the stress response (Fig. 4f).

## Discussion

In the present study using the microinjection technique targeting RVLM, we compared response to Glu among WKY, SHR and SHRSP. The results indicated that the response was significantly greater in SHRSP than in the other two. As the difference was abolished with losartan or tempol, AT1R and ROS production seemed involved in the exaggerated response in SHRSP. Further, the evaluation in the P22PHOX-depleted SP.MES implied that an exaggerated NOX activity was responsible for the enhanced response in SHRSP. Telemetry experiments further suggested that the NOX system as well as AT1R activation in the brain was likely to contribute to an exaggerated sympathetic response to cold stress in SHRSP.

Although a previous report compared the response to Glu microinjection between SHR and WKY^14^, no reports were so far available on the comparison between SHR and SHRSP. The present study characterized SHRSP as a hypertensive animal model with a greater ROS level and an exaggerated sympathetic response to Glu even when compared with SHR that share a similar genetic background.

In this study, SP.MES provided a new insight into a putative pathophysiological role of NOX in the exaggerated sympathetic response observed in SHRSP. P22PHOX is a subunit essential for the activity of the NOX complex^3^; among four major subtypes of NOX expressed in the cardiovascular system, three (i.e., NOX1, 2 and 4) are known to require P22PHOX for their activity^3^. Accordingly, SP.MES was expected to have a low NOX activity, which was indeed shown in the lucigenin experiment and DHE staining (see Fig. 3 and S1). Of interest, the ROS production in SP.MES was comparable with that in SHR, implying that the excess level of ROS in SHRSP was mainly due to the P22PHOX-dependent NOX activity.

Under such condition, SP.MES showed an attenuated response to the Glu microinjection when compared with PM0 (this reference strain was basically the same as SHRSP; see Materials and Methods). Apocynin as well as tempol reduced the exaggerated BP response in PM0 to a level comparable with that in SP.MES, and further, no additional reduction was observed when these reagents were administrated in SP.MES (see Fig. 2). All of these observations implied that a higher level of ROS in SHRSP due to the higher NOX activity, causally related to the exaggerated response to Glu. This interpretation was supported by the observation that microinjection of diethyldithiocarbamate (DETC), an inhibitor for the superoxide dismutase that could increase ROS level, in RVLM induced higher response to Glu (see Fig. S2).

Previous studies indicated that AT1R activation stimulated ROS production in different tissues including the brainstem^3^,^12^,^13^,^15^. As losartan reduced the response to Glu not in SP.MES but in SHRSP in this study, it was speculated that AT1R activation potentiated the response in SHRSP through ROS production. This possibility should be tested.

In previous studies on hypertensive model rats, AngII in CNS was implicated in sympathetic stress responses through ROS production^12^,^13^,^15^. In accordance with them, the present study showed that, in SHRSP, losartan reduced the BP response to Glu when microinjected into RVLM (see Fig. 1). As AngII was not exogenously injected into RVLM in this experiment, the observed effect of losartan inferred endogenous AngII in RVLM of SHRSP stimulating AT1R in the baseline condition. Although no firm evidence is available about the origin of endogenous AngII in RVLM, some histological studies indicated angiotensinergic neurons found in RVLM^16^,^17^. It is of interest to examine the role of angiotensinergic neurons in RVLM in SHRSP in future studies. SHRSP was repeatedly indicated to be sensitive to stress^18^,^19^,^20^ and the ROS-dependent enhancement of Glu response might be responsible for this phenotype in SHRSP. This was supported by the observation that BP response to cold stress were greater in SHRSP, which was attenuated either by losartan infusion into the lateral ventricle or by the genetic depletion of the NOX activity (i.e., in SP.MES).

Table 1 and Fig. 4 showed that P22PHOX-depleted SP.MES had a significantly lower BP than that in SHRSP. This implied that P22PHOX-dependent NOX activity was important in the pathogenesis of hypertension in SHRSP. In this context, it is of interest that Weber *et al*. showed that a transgenic mouse overexpressed P22PHOX only in smooth muscle was hypertensive as well as had hypertrophy in the aortic wall only when treated with Ang II^6^. This observation implied that P22PHOX-dependent NOX activity had particular importance in the pathogenesis of Ang II-induced hypertension. In SP.MES, P22PHOX was depleted in all types of cells, and thus pathological reaction to Ang II might be reduced not only in the sympathetic nervous system but also in vascular smooth muscle, resulting in low BP. It may be interesting to study biological differences of smooth muscle cells between SHRSP and SP.MES.

This study has several limitations. First, as the putative congenic region including *Cyba* was estimated 1.4 Mbp or less, SP.MES harbored up to 30 genes of the MES allele located in this region (see Table S1). Accordingly, we cannot genetically exclude a possibility that other genes in the transferred genomic fragment conferred phenotype changes found in SP.MES. In spite of that, as a significant decrease in the ROS level in SP.MES in parallel with changes in BP was shown in the present study, the mutation in the *P22phox* gene was the most probable genetic change responsible for the phenotypes in SP.MES. Second, as several members of the NOX family are known to require P22PHOX, it was not possible to tell which subtype(s) of NOX was responsible for this exaggerated sympathetic response. Although apocynin was once suggested to be specific to NOX2, several reports showed evidence against it^10^,^11^. Among subtypes of NOX, it is known that NOX2 and 4 are most abundantly identified in the brain^3^. While NOX2 is a prototype of NOX generating ROS, NOX4 was recently shown to play a protective role in the vasculature^21^. However, since this has not been proved yet in CNS especially in terms of sympathetic regulation, it may be necessary to wait for NOX2- and NOX4-depleted rats by genome editing technology to evaluate the role of these NOX subtypes in RVLM.

In conclusion, we showed that the response to Glu microinjection into RVLM was significantly greater in SHRSP than in SHR and WKY, which seemed to depend on a higher level of ROS in this strain. The observation in SP.MES suggested that the exaggerated response to Glu as well as high ROS level in SHRSP was due to P22PHOX-dependent NOX activity. As SP.MES is equivalent to SHRSP except lack of functional P22PHOX, this congenic strain is useful model to study roles of the NOX system in hypertension and hypertensive organ damages when used in combination with SHRSP.

## Materials and Methods

### Construction of the congenic SHRSP.MES-(*Cyba*)/Izm

SHRSP/Izm and WKY/Izm were distributed by the Disease Model Cooperative Research Association (Kyoto, Japan). MES rats were obtained from SLC (Hamamatsu, Japan) under an agreement of Dr. Kiyoshi Matsumoto. MES harbored a 51 bp-insertion in the transcript of *P22phox* due to splicing abnormality, which resulted in the loss of the protein expression^8^. A congenic strain, SP.MES, was developed using the marker-assisted ‘speed congenic’ strategy^8^,^22^. A hundred and forty-one simple sequence repeat markers located throughout the whole genome were employed in the selection at each generation. The genotype of *Cyba (P22phox*) was checked by PCR as described in the previous report^23^. After five generations of backcrossing, the genomic fragment between D19Rat21 and D19Rat105 including *Cyba* (the maximal size was 1.4 Mbp) was confirmed to be transferred from MES to SHRSP, and brother-sister mating was performed to establish the congenic SP.MES homozygous for the MES allele of *Cyba* (Fig. S3a). The homozygote for the wild (i.e., SHRSP type) allele of *Cyba* was simultaneously obtained, and used as a control strain for SP.MES (named PM0). All the other markers examined were confirmed as homozygous for the SHRSP allele in both SP.MES and PM0. The 51-bps insertion in the transcript and the lack of protein expression of P22PHOX was confirmed in SP.MES by RT-PCR and Western blotting (Fig. S3b and ref. 7). Since P22PHOX is an essential subunit of the active NOX, the ROS level was expected to be low in SP.MES, and indeed, DHE staining (see below for the method) showed a lower level of superoxide in RVLM in SP.MES (Fig. S1).

### Animal

The current study was in accord with and approved by the Institutional Review Boards of the local committee of animal research in Shimane University. The committee gave written consent and procedures how to include animals in the study. The studies were performed in accordance with the relevant guidelines. Male rats at 11–12 weeks of age were used in the experiments. All the rats were fed the stroke-permissive diet (Funabashi Farm Co. Ltd, Chiba, Japan) and water *ad libitum*. Systolic blood pressure (SBP) was measured in each conscious rat using the tail-cuff method (BP-98A; Softron Corp., Tokyo, Japan). The average of five measurements was used as a representative SBP of an individual rat

### RVLM microinjection

Rats were anesthetized by intraperitoneal injection of pentobarbital (50.0 mg/Kg BW). The femoral vein was cannulated and connected to an auto-injector (ESP-32, Eicom, Japan) for continuous infusion of pentobarbital to control anesthesia throughout the entire experiment (20.0 mg/kg/h). Then the femoral artery was cannulated and connected to a pre-calibrated transducer (AD Instruments PowerLab 8/30, Australia) to monitor intra-arterial BP. The trachea was intubated for artificial respiration. Rat’s head was fixed at the horizontal plane in a stereotaxic frame (Narishige Scientific Instrument Lab., Tokyo, Japan), and the skull was opened and unilateral RVLM microinjection was performed with a glass micropipette (tip diameter, 50 μm); microinjection volume was 100 nL and injection period was 10 s. RVLM was located 12 mm caudally to the bregma, 1.9 mm lateral to the midline and 8 mm ventral to the surface of the cerebellar vermis^24^. The location of RVLM was confirmed functionally by the pressor response (≥20 mm Hg) to microinjection of 2 nmole of Glu^25^. The micropipette was then removed and rinsed thoroughly with physiological saline. After at least 10-min interval, 5 nmole Glu, 1 nmole 4-hydroxy-2,2,6,6-tetramethylpiperidin-1-oxyl (tempol)^2^, 1 nmole losartan^15^ and 1 nmole apocynin^9^ were injected according to the protocol. When inhibitory effects of chemicals were examined, Glu was injected 1–2 min after the injection of the chemicals tested. An averaged SBP for at least 10 min just before the injection was obtained as baseline SBP, i.e., the pressure measured at the resting condition without any stressor effect. The maximum change in SBP was taken to represent the response to the injection. India ink was injected at the end of some experiments to confirm the location of injection point (Fig. S4).

### Measurement of the superoxide level in the brainstem

Superoxide production was quantified by the lucigenin-enhanced chemiluminescence as described previously^26^. In brief, a slice including RVLM at approximately 2 mm thick of the brain stem was collected and used in the lucigenin assay and the DHE staining. The brainstem (mostly ventrolateral medulla) was dissected from rats euthanized with pentobarbital and homogenized in a 20 mM sodium phosphate buffer (1 gm tissue per ml buffer), pH 7.4, containing 0.01 mM EDTA. The supernatant was recovered immediately after a low speed centrifuge at 1000 g for 10 min at 4 °C to remove nuclei and unbroken cell debris and used to measure for superoxide anion. Light emission produced by reaction of lucigenin (5 μM) with superoxide was measured with a luminometer (LB 9506, Berthold Technologies, Bad Wildbad, Germany) for 30 min at room temperature. Superoxide production was expressed as relative light unit (RLU)/min/ μg protein. The protein concentration was determined using the Protein Assay Bicinchoninate kit (Nakalai tesque, Kyoto, Japan).

Dihydroethidium (DHE) staining was done by the method of Schupp *et al*.^27^. In brief, cryosections (5 μm) of brainstem at the level of RVLM were stained with DHE (10 μmol/L) for 30 min at room temperature in the dark. Serial sections were treated with NADPH (100 mmol/L) and apocynin (10 μmol/L) for 30 min before incubation with DHE. Photographs were taken with the DS-Ri1-U2 microscope (Nikon, Japan).

### Response to cold stress

Response to cold stress was evaluated as described previously^28^. A telemeter probe (TA11PA-C40; Data Sciences International) was implanted into rats under anesthesia with pentobarbital (50 mg/Kg BW, i.p.) and the rats were kept for one week for recovery. Baseline BP was recorded over 3 consecutive days. Then, cold stress was applied for 3 h and changes in SBP were recorded every 10 min during the experiment. To measure u-NE, rats were placed in metabolic cages for 6 h to collect urine at a room temperature. After one day interval, urine samples were again collected for 6 h at 4 °C. One ml of 1.0 N HCl was added to collection tubes to avoid degradation of norepinephrine. Urine was centrifuged at 1000 rpm for 10 minutes at 4 °C and stored in aliquots at −80 °C until quantification by HPLC. All experiments using rats were conducted in the afternoon during the light phase.

In the experiments infusing losartan into the lateral ventricle, a telemetry probe was implanted in a male SHRSP at 8 weeks of age. After one week of recovery, cannulation into the left lateral ventricle was performed, which was connected with a programmed micro-infusion pump (iPRECIO, Primetech Corp. Tokyo, Japan) implanted under the skin. Artificial cerebrospinal fluid (aCSF) (122 mM NaCl, 25 mM NaHCO_3_, 3.1 mM KCl, 1.3 mM CaCl_2_, 1.2 mM MgSO_4_, 0.4 mM KH_2_PO_4_, and 10 mM D-glucose, pH 7.4) was then continuously infused at 2.5 μL/h. After a recovery period of 3 days, baseline SBP was recorded for 2 consecutive days. Stress-induced SBP elevation was then measured at 4 °C for 3 h. Subsequently 2.4 mg/mL losartan (dissolved in aCSF) was substituted for aCSF, which was infused at a rate of 2.5 μL/h (6.0 μg/h losartan) for 2 days^12^. SBP at room temperature and at 4 °C were then recorded as described above. SBP was averaged for 1 h before and 3 h after the start of cold stress to represent SBP at room temperature and under the cold stress, respectively.

### Chemicals

Glu was purchased from Wako (Tokyo, Japan). Tempol, DETC, apocynin, NADPH, lucigenin and DHE were purchased from Sigma Aldrich (St. Louis, MO, USA). Losartan was generously provided by MSD Corp (Kenilworth, NJ, USA). All chemicals were dissolved in physiological saline except apocynin that was dissolved in hot distilled water. The pH of all solutions was adjusted to be 7.4.

### Statistics

All values were expressed as mean ± SD. Statistical analyses were performed either using the Bonferroni’s post-hoc test or the Student’s *t* test when they were appropriate (see the figure legends). The Dunnett’s post-hoc test was used in Table 1. *P *< 0.05 was considered to be significant.

### Ethical Approval

Ethical concerns and experimental protocols were approved by the local committee of animal research in Shimane University.

## Additional Information

**How to cite this article**: Zahid, H. M. *et al*. Effect of *p22phox* depletion on sympathetic regulation of blood pressure in SHRSP: evaluation in a new congenic strain. *Sci. Rep.* **6**, 36739; doi: 10.1038/srep36739 (2016).

**Publisher’s note:** Springer Nature remains neutral with regard to jurisdictional claims in published maps and institutional affiliations.

## Supplementary Material

Supplementary Information

## Figures and Tables

**Table 1:** Blood pressure and body weight of the strains.

strains (N)	BP, mmHg	BW, g
SHRSP (10)	220 ± 13*	269 ± 21
PM0 (11)	212 ± 11*	284 ± 15
SP.MES (11)	188 ± 13	231 ± 12*
WKY (11)	117 ± 4*	341 ± 18*
SHR (7)	185 ± 7	288 ± 11

Blood pressure (BP) and body weight (BW) were measured at 12 weeks of age. *Significantly different from the value of SHR (Dunnett’s post hoc test).
