# Supplementary material for: Effect of p22phox depletion on sympathetic regulation of blood pressure in SHRSP: evaluation in a new congenic strain
Source: Sci Rep. 2016 Nov 8;6:36739. doi: 10.1038/srep36739 (PMC5099856; doi:10.1038/srep36739)
Supplement: Supplementary Information [file srep36739-s1.pdf]

Supplementary Information

Effect of *p22phox* depletion on sympathetic regulation of blood pressure in SHRSP: evaluation in a new congenic strain

Hasan M. Zahid*, Mohammed Zubaerul Ferdaus†, Hiroki Ohara, Minoru Isomura, Toru Nabika

Department of Functional Pathology, Shimane University School of Medicine, Izumo, Japan.

*Correspondence to

Hasan M. Zahid

Department of Functional Pathology, Shimane University School of Medicine,

Izumo 693-8501, Japan. Tel: +81-853-20-2136, Fax: +81-853-20-2135

E-mail: hasan@med.shimane-u.ac.jp

†: the present address of MZF is Division of Nephrology and Hypertension, Department of Medicine, Oregon Health and Science University, Portland, OR, OR97239, USA

Supplementary Method:

Staining:

For histological assay, hematoxylin and eosin (H&E) staining was performed as previously reported method[1]. Briefly, samples were collected from the brain stem and perfused with 1X PBS then fixed and stored in 4% paraformaldehyde. The samples were then frozen in Tissue-Tek OCT (Sakura Finetek, Torrance, CA, USA) and stored at -80°C. Coronal cryosections (10 μm) were performed using a cryostat (CM3050S, Leica, Japan) and stained with HE. Photographs were taken with the DS-Ri1-U2 microscope (Nikon, Japan).

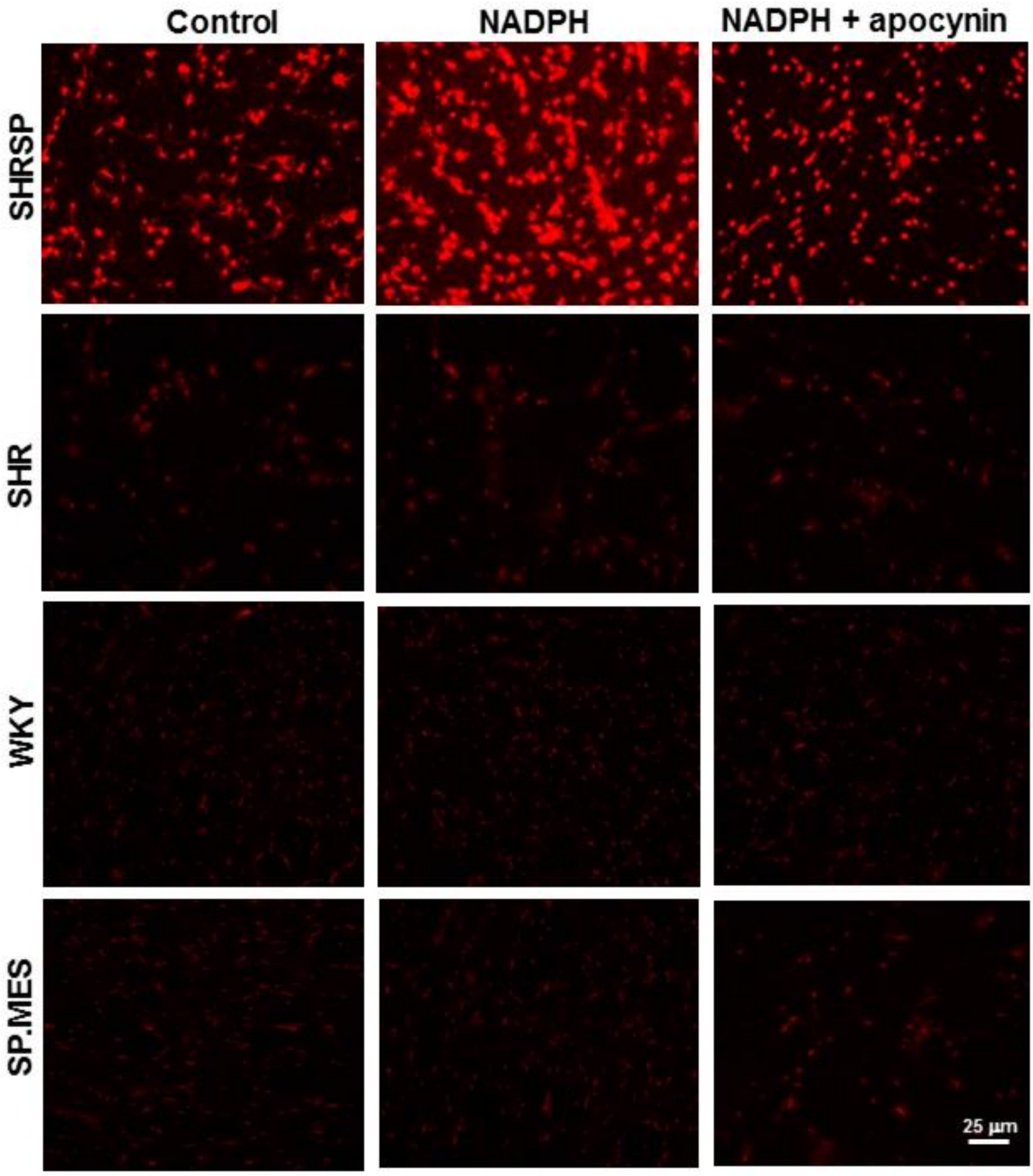

Figure S1: ROS level in the brainstems by DHE staining.

Tissues collected from the brainstems were sliced (5 µm) and stained with dihydroethidium (DHE, 10 µmol/L). 20X magnification of the oxidative red fluorescence was analyzed by fluorescent microscope, DS-Ri1-U2 (Nikon, Japan). Scale bar, 25 µm. The representative photographs are shown for each set of treatment in every strain.

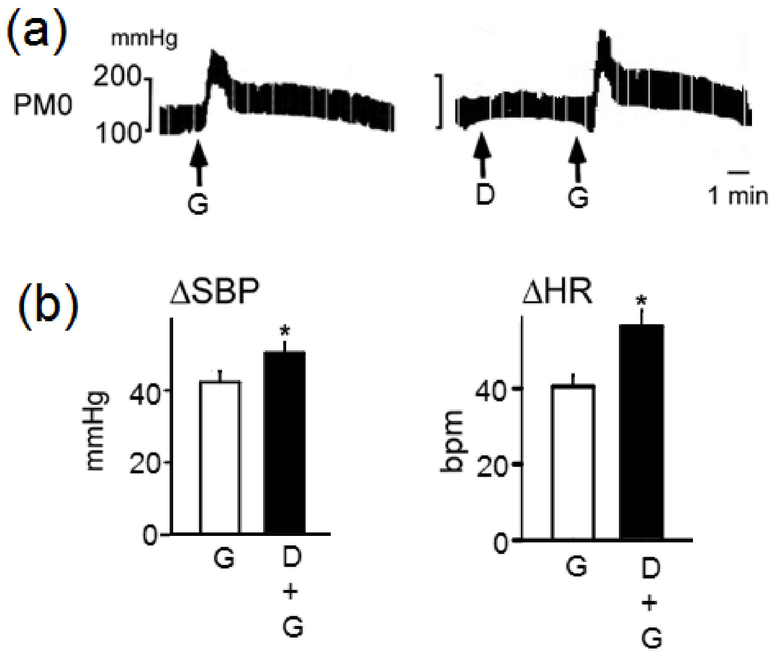

Figure S2. Effect of DETC on response to Glu

(a) Representative recordings of effects of DETC (D) (10 nmole) on the response to Glu (G).

(b) The summary of the response data. Seven PM0 were used in the experiment.

Data on Glu alone were the same as those in Fig. 2. *; significantly different from the response to Glu alone (by the Student’s t-test).

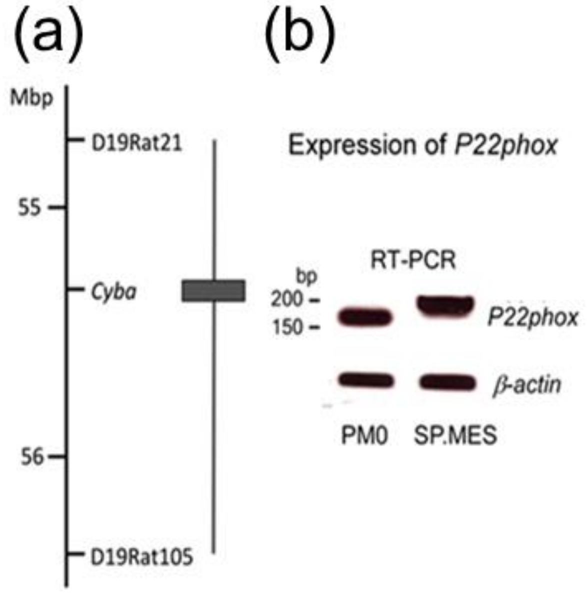

Figure S3. Basic characters of SP.MES

(a) The congenic region of SP.MES. The black box indicates a chromosomal fragment transferred from the MES rat to SHRSP. The vertical bar indicates the region including recombination points. (b) The abnormal insertion in the *P22phox* transcript identified by RT-PCR.

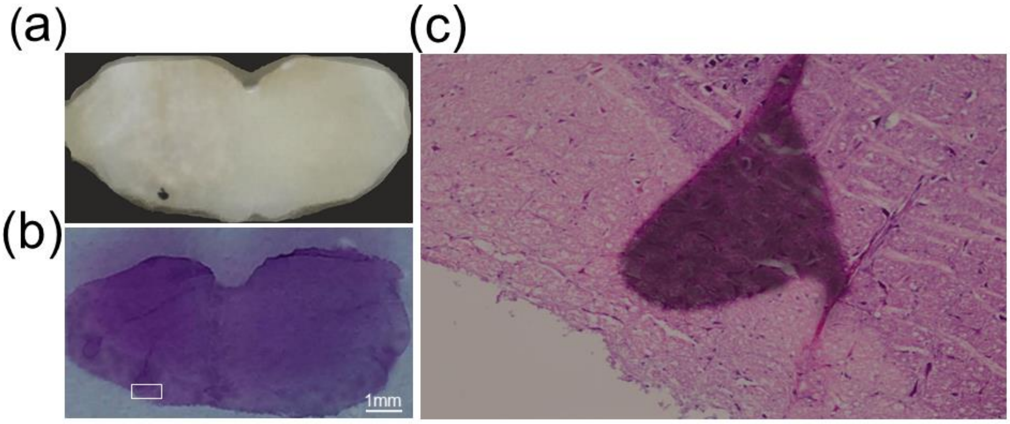

Figure S4: Histological verification of the injection site.

India ink was injected after RVLM was located by glutamate microinjection (see Methods). (a) cut surface at RVLM in the brainstem (b) after staining with hematoxylin and eosin (c) 20X magnification of the area indicated with a box in (b). Scale bar = 1 mm. The indicated RVLM location was -11.8 mm far from the bregma. Microinjection sites were referred to standard anatomic structures of the brain stem according to the atlas of Paxinos and Watson[2].

Table S1. List of genes located in the 1.4-Mbp congenic fragment in SP.MES

Symbol of the genes	putative functions
Car5a	Hydration of carbon dioxide
Banp	Tumor suppressor and cell cycle regulator
Zfpm1	Transcription regulator in cell differentiation
Trhr2	Participates in calcium/calcium-mediated signaling pathway
Zc3h18	Acts as zinc finger protein
Il17c	Protection against pathogens
P22phox (Cyba)	Forms active NOX complexes and produces ROS
Mvd	Biosynthesis of isoprenes
Snai3	Inhibits myoblast differentiation
Rnf166	Regulate many different cellular functions
Ctu2	tRNA modification
Fam38a	Associated with breast cancer
Cdt1	DNA replication factor
Aprt	Role in purine nucleotide salvage pathway
Galns	Degradation of the glycosaminoglycans, keratan sulfate, and chondroitin 6-sulfate
Trappc2l	Involves in protein trafficking
Pabpn1l	Binds the poly A tail of mRNA
Cbfa2t3	Breast tumor suppressor
Cdh15	Control morphogenetic process
Spg7	Regulation of cell components and proteins
Rpl13	Involves in ribosome structure and RNA binding
Cpne7	May function in membrane trafficking

Sult5a1	Transfer of sulfur from biological sulfur donor
Dpep1	Renal metabolism of glutathione and its conjugates, hydrolysis of the β-lactam ring of antibiotics
Chmp1a	Multivesicular body sorting of proteins to the interiors of lysosomes
Spata33	Associated with spermatogenesis
Cdk10	Role in cellular proliferation
Spata2L	May have a role in the regulation of spermatogenesis
Vps9d1	Acts as transporter and GTPase activator
Fanca	Role in fanconi anemia pathway

Source: http://www.ncbi.nlm.nih.gov/

References:

1. Lagraoui, M. *et al.* Controlled cortical impact and craniotomy induce strikingly similar profiles of inflammatory gene expression, but with distinct kinetics. *Front Neurol* **3**:155 (2012).

2. Paxinos, G. & Watson, C. The Rat Brain in Stereotaxic Coordinates. *NY: Academic Press* 2nd ed. New York (1986).
